# Evaluation of an Australian Health Literacy Program Delivered in Adult Education Settings

**DOI:** 10.3928/24748307-20190402-01

**Published:** 2019-10-03

**Authors:** Kirsten J. McCaffery, Suzanne Morony, Danielle M. Muscat, Andrew Hayen, Heather L. Shepherd, Haryana M. Dhillon, Sian K. Smith, Erin Cvejic, Wedyan Meshreky, Karen Luxford, Don Nutbeam

## Abstract

**Background:**

Adult education targeting health literacy (HL) may bring added value in the form of improved health.

**Objective:**

This study evaluated the effects of a HL program as part of an adult education curriculum for adults with low literacy and numeracy.

**Methods:**

This was a partial-cluster randomized controlled trial among 308 adults enrolled in basic education programs in Australia. Of the 308 participants, 141 (46%) were randomized to either the standard program (language, literacy, and numeracy [LLN]), or the HL intervention (LLN with embedded health content); the remainder (*n* = 167) were allocated to standard intervention programs by the education provider at the class level. The main outcomes were functional HL, self-reported confidence, patient activation, generic HL (ie, HLQ, health knowledge, and self-reported health behavior). Data were collected at baseline, immediately after, and at 6 months post-intervention.

**Key Results:**

Of the 308 participants, 71% had limited literacy and 60% spoke a language other than English at home. Both interventions benefited participants, with improvements from baseline to immediate follow up on individual-level functional HL (e.g., reading a thermometer; HL group 18.4% vs. standard group 7.2%; *p* = .001), confidence (HL group 0.34 vs. standard group 0.06; *p* = .014) and health literacy questionnaire (HLQ) subscales. At 6 months, improvements in confidence (*p* < .001) and some HLQ measures were retained. A consistent pattern of increased improvement in the HL program was observed compared to the standard program, although only some measures reached statistical significance: reading a food label (HL group 6.03/10 correct vs. standard group 5.49/10 correct; *p* = .022); confidence (*p* = .008); ability to actively manage health (HLQ) (*p* = .017), and health knowledge at 6 months (HL group 68% vs. standard group 60% correct, *p* = .052). HL participants reported being more likely to share course information and rated the program more useful to understand their health.

**Conclusions:**

Improving language, literacy, and numeracy generally has potential public health benefits that are retained at 6 months. Integrating health content adds further value to adult basic learning, is feasible, and potentially scalable. **[*HLRP: Health Literacy Research and Practice*. 2019;3(Suppl.):S42–S57.]**

**Plain Language Summary:**

We compared the effect of an adult education-based health literacy (HL) program versus a standard language, literacy, and numeracy program on students' HL skills and psychosocial outcomes. Although students in both trial arms improved their skills, students in the HL program had better outcomes with higher HL, greater confidence, and higher health knowledge scores at 6 months.

Health literacy is defined as the capacity to acquire, understand, and use information in ways that promote and maintain good health (U.S. Department of Health and Human Services, 2019; U.S. Department of Health and Human Services, 2000), and it has a well-established association with health outcomes independent of other known risk factors (Berkman, Sheridan, Donahue, Halpern, & Crotty, 2011). From an asset-based perspective, health literacy can be seen to be developed through education to support greater engagement and autonomy in health decision-making (Nutbeam, McGill, & Premkumar, 2017); “functional health literacy” describes basic level skills sufficient for people to obtain relevant health information and be able to apply that knowledge to a range of prescribed activities in everyday situations; “communicative/interactive health literacy” includes the more advanced skills to extract information, discriminate between sources of information, and derive meaning; and “critical health literacy” includes the ability to obtain information from a variety of sources, critically analyze this information, and make informed decisions (Nutbeam, 2000; Smith, Trevena, Nutbeam, Barratt, & McCaffery, 2008). This delineation derives from mainstream literacy studies (Freebody & Freiberg, 1997; Freebody & Luke, 1990; Nutbeam, 2000) and is valuable because it signals the differences in skill levels able to support and affect health-related decisions and behavior.

Extensive research shows that adults with limited health literacy skills are less able to engage in preventive health-care, are more likely to develop chronic illnesses (Bennett, Chen, Soroui, & White, 2009), experience greater difficulties managing illnesses, and have higher rates of mortality (Berkman et al., 2011). The cost of low health literacy to the health system is also considerable, with data from the United States suggesting an additional 3% to 5% of total cost incurred per year among adults with limited health literacy (Eichler, Wieser, & Brugger, 2009). National population survey data show that low health literacy is widespread. In Australia, up to 60% of adults lack basic skills to understand health-related materials, such as instructions on a medicine label (Australian Bureau of Statistics, 2006). Estimates of low health literacy in Europe (47%) (World Health Organization, 2013.) and the U.S. (36%) (Kutner, Greenberg, Jin, & Paulsen, 2006) are similarly high. Improving health literacy in populations is a priority in Australia and internationally (Australian Commission on Safety and Quality in Health Care, 2014; World Health Organization, 2013).

## Health Literacy Interventions

Health literacy can be improved through the provision of information, effective communication, and structured education, as well as by changes to the organization and delivery of health services. Improvements in health literacy can be assessed through the measurement of changes to the knowledge and skills that enable well-informed and more autonomous health decision-making. To date, most research into health literacy has focused on the development of effective interventions to increase functional health literacy in clinical practice. There are relatively few reported interventions with community populations to improve higher-level interactive and critical health literacy skills. A recent review of interventions with adult community populations found few actively using the concept of health literacy in their design and evaluation (Nutbeam et al., 2017). Those identified covered a range of settings, including online programs, adult education, and maternal, infant, and early childhood home-visiting programs. Educational methods varied considerably from formal classes, home visiting, and study circles, to multimedia and eHealth/online interventions (Nutbeam et al., 2017).

Partnerships between health and education agencies offer promise in building health literacy in community populations (Chen, Goodson, & Acosta, 2015; U.S. Department of Health and Human Services, 2019; Martinez et al., 2013; Santos, Handley, Omark, & Schillinger, 2014; Sholet, 2002; Simons-Marton, McLeroy, & Wendel, 2012), especially if they collaborate to access hard-to-reach groups and develop strategies to improve both health literacy and general language, literacy, and numeracy (LLN) skills and English as a second language (ESL) (Chen et al., 2015; Nutbeam, 2008; Wickert & McGuirk, 2005). Although there have been some interesting partnerships between the fields of health and adult education internationally and recognition that there is value in bringing both fields together, there has also been concern about the appropriateness of asking adult educators to teach health content because this is not the area of their expertise (Rudd, 2002). In Australia, there have been few partnerships between adult literacy and the health sector (Black, 2012; Green, Bianco, & Wyn, 2007), and recent political and budgetary changes have forced adult education to move toward a strong exclusive focus on vocational skills (Wheelahan, 2015).

One notable successful community-based program that embedded health content into a general adult basic education in the United Kingdom is the “Skilled for Health” program (Tavistock Institute and Shared Intelligence, 2009). This national government-funded initiative aimed to engage people with low skills in learning to improve their health and life skills. It was comprised of a curriculum of teaching and learning materials on health improvement topics that embedded literacy and numeracy skill development across a range of topics such as healthy food and drink, physical activity, finding out about health concerns, and self-care. The program was designed to be run in a variety of adult-learning group settings such as the workplace, migrant and other vulnerable community groups, and prisons. Using a pre-/post-evaluation design, the study evaluation reported that health content helped engage and retain adult learners who are socially disadvantaged and that participants demonstrated improved understanding about health and self-reported healthier behaviors (diet, exercise, and smoking). However, although the program was delivered successfully to more than 17,000 adult learners and considered to be effective, the evaluation was limited by its use of a nonrandomized design and reliance on self-reported outcome measures. (Tavistock Institute and Shared Intelligence, 2009).

### The Australian Health Literacy Program

We developed a health skills education program for delivery through an established adult basic skills program offered through government-funded Technical and Further Education (TAFE) colleges in New South Wales (NSW), Australia. The program was based on the UK Skilled for Health program (Tavistock Institute and Shared Intelligence, 2009) with the aim to improve participants' health literacy. The program used functional context education methods (an approach to adult learning that embeds functional basic skills within topics that are of relevance and interest to adult learners) with the aim of maximizing students' motivation and persistence to enhance learning (Sticht, 2000). The Australian program had similar content and design but was adapted for the Australian context, using Australian health examples and materials, commonly used language, and health topics related to Australian national health priorities. We also added a new topic on shared decision-making that was designed to teach students how to participate in health decisions with their health care professionals (McCaffery et al., 2016). This was designed to build communicative and critical health literacy skills on top of functional health literacy. The program was designed to be delivered in TAFE colleges by adult basic education practitioners.

The current study aimed to compare the effects of the health literacy education program to a standard basic literacy and numeracy program operated at adult education centers in Australia. These basic literacy and numeracy programs are designed to develop LLN skills in the numerous contexts in which people work, learn, and communicate that may also lead to important improvements in health (McLean, Perkins, Tout, Brewer, & Wyse, 2012). This study sought to compare the impact of the two interventions to deliver improvements in health literacy, confidence, knowledge, and health behavioral outcomes.

## Methods

### Study Design

**Figure 1.**
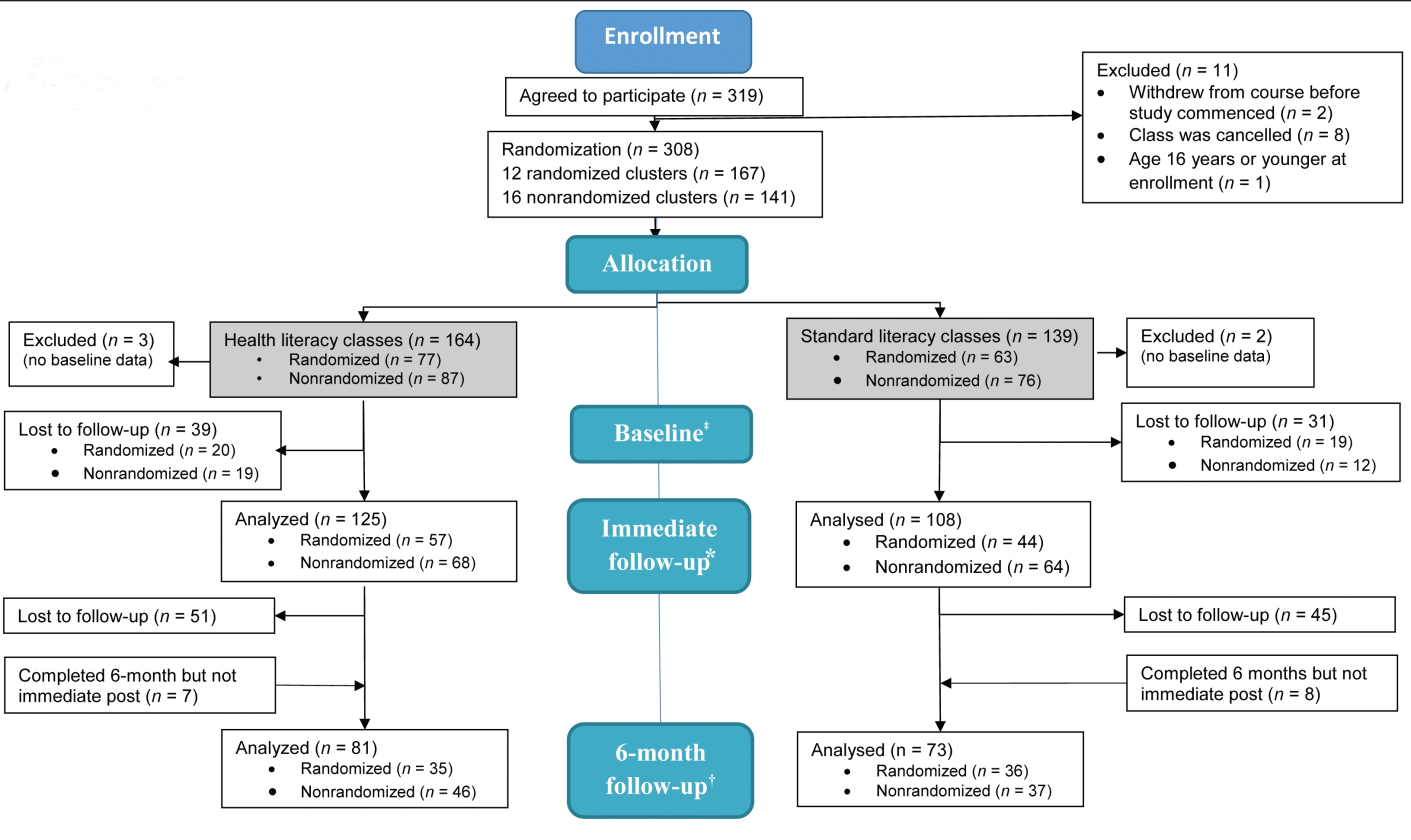
Flowchart showing design of the study. ^‡^Completed at least one (of three) baseline booklets. *Reasons for loss to immediate follow-up (course dropout or absence during data collection sessions) include sickness, overseas travel, gained employment. ^†^Reasons for loss to 6-month follow-up include changed address, difficulty completing forms.

We conducted a (partially) matched cluster-randomized controlled trial in which matched groups (intervention/standard LLN] are pairs of classes with similar demographic characteristics at participating TAFE sites across NSW (**Figure 1**). Students were enrolled in Level 2 (basic/beginner) classes according to the 5-level Australian Core Skills Framework (McLean et al., 2012). These classes typically contain a mix of adults with variable literacy and language needs such as recent refugees and migrants needing to develop their English language skills, as well as native-born Australians with low educational attainment and those who are unemployed and required by Centrelink (the national Australian unemployment service) to attend classes to remain eligible to receive benefits.

Students were invited by their teacher to participate in a research study and gave written informed consent. Consent from a parent or guardian was obtained for students younger than age 18 years at time of enrollment in the study. The University of Sydney Human Research Ethics Committee (ID# 2013/938) and each participating TAFE Institute approved the trial. The published protocol (McCaffery et al., 2016) describes the design in detail.

### Participants

Students (age 16 years and older) graded by their teachers as Level 2 learners using the Australian Core Skills Framework and teachers from participating TAFE colleges in NSW (see Appendix 1, available online at http://hdl.handle.net/2123/20364).

### Randomization and Masking

At enrollment, students at the same site were randomized into matched classes when possible using simple techniques. Students selected a concealed number from an envelope that randomly assigned them to the intervention or standard class. Students who had already been allocated to classes prior to joining the study (e.g., classes who had already been working with the same teacher in a prior semester, or classes at different sites) were randomized at the class level centrally at the University of Sydney by the research team. Some TAFE sites indicated they would not participate in the study if they could not select which classes received the intervention and were included in the study sample. In some cases, this was due to a strong preference for the health content, and for others because teachers felt the standard program was needed for students to focus on employment-related skills such as improving computer literacy. In all cases, teachers and program coordinators emphasized their ethical responsibility to teach to their students' needs.

Students were blinded to the intervention in so far as they were not informed that the purpose of the study was to evaluate a health literacy program. A written debrief statement was distributed to all students after the final data collection described the purpose of the trial (McCaffery et al., 2016) either by hand (via their teacher) or by mail.

### Curricular Intervention

Development, piloting, and refinement of the intervention is described in detail elsewhere (McCaffery et al., 2016; Muscat et al., 2017). Australian adult education and public health experts adapted 29 topics from the UK Skilled for Health program for an Australian context. Using functional context education methods (an approach to adult learning that embeds functional basic skills within topics that are relevant and of interest to adult learners) (Sticht, 2000), we embedded key LLN skills development into learning materials that focused on Australian national public health priorities. The original “Skills for Health” program included more than 60 topics that covered two broad themes: (1) health promotion and well-being, and (2) accessing services and self-care. For the Australian program, we added a new topic on shared decision-making described in Muscat et al. (2015) and Muscat et al. (2019). Students were required to study 10 core subjects but then were able to select from the remaining 19 topics. This supported a learning environment in which teachers could tailor the program to areas of high interest for students, and students themselves could make meaningful choices about content. The “real world” nature of the program, using everyday examples, real scenarios, and content, was also designed to support transferability of learned skills to new tasks. For example, the course embedded basic reading (e.g., reading an immunization program schedule), writing (e.g., labelling the human body), speaking (e.g., participating in doctor-patient consultation role plays), listening (e.g., listening to simulated emergency phone calls), and numeracy (comparing units of measurement) (Morony et al., 2018).

**Table 1** lists the topics covered by the health literacy program. Teachers were given a suggested delivery plan, with advice that they may diverge from this according to the interests and capabilities of their students. The main requirement for delivery was that classes cover all core topics considered central to the health literacy learning objectives and linked to the quantitative assessments. Teachers varied in how they delivered the program; some described attempting to cover all topics whereas others focused on fewer topics selected by their students (Muscat et al., 2017).

All classes were taught in English. The composition of the classes varied depending on the location of the TAFE. Some sites had predominantly native English-speaking students, whereas others had students from a wide range of different cultural and linguistic backgrounds, reflecting the migrant diversity across NSW. All course content was reviewed by Australian health experts (including partner organizations - NSW Ministry of Health, National Prescribing Service, Medicine Wise, and NSW Clinical Excellence Commission) (see Appendix 2, available online at http://hdl.handle.net/2123/20364).

*The standard LLN program.* The standard program was run for the same length of time and hours as the intervention in all matched and randomized classes. Typically, classes were run twice per week for between 2 and 4 hours but this varied in different sites. The LLN program followed the standard TAFE content for the units of study. This included learning computer skills, employment skills, and other nonhealthy-related activities. The LLN program is designed to develop core skills and confidence in language and improve functional English LLN skills. Teachers following the standard program were requested not to cover health content.

### Procedures

All classes were delivered by experienced TAFE teachers who were trained in the study procedures at a 1-day workshop. Demographic data were captured on the TAFE enrolment form and the study questionnaire. Assessments at baseline (start of the semester prior to intervention) and immediate follow-up were divided into up to three sections and delivered in the first three classes of the course. This was to reduce the cognitive and time burden on students, which was onerous at the start of the program. The questionnaires were designed to be completed by the students independently; however, the teacher was available to provide assistance with reading questions when required. The 6-month follow-up questionnaire was completed in a 1-hour session at a TAFE site, or by mail or telephone. Students were initially invited to attend the session, and those who did not attend were sent the questionnaire through the mail to return or to complete over the telephone with the study researchers. All participants who completed the 6-month assessment received a $20 gift voucher.

Data were collected on paper-based questionnaires and entered into a central computerized database. Ten percent of records were manually checked against the paper copies. Knowledge items were coded as correct against marking criteria developed by the research team and double-checked by two researchers to ensure accuracy and consistency (McCaffery et al., 2016).

### Measures

*Demographic and health measures (assessed at baseline).* The demographic and health measures included age, sex, country of birth, language spoken at home, and self-reported health status. Health literacy measures included the Single Item Literacy Screener “How often do you need to have someone help you when you read instructions, pamphlets, or other written material from your doctor or pharmacy?” (Morris, MacLean, Chew, & Littenberg, 2006); self-report of reading ability “How would you rate your ability to read?” (Jeppesen, Coyle, & Miser, 2009), using a cut off of 3 (“sometimes/okay”) to denote poor literacy; and Newest Vital Sign (NVS) (Weiss et al., 2005) with minor modification to the food units presented in the stimulus to make it suitable for use in Australia.

*Outcome measures (assessed immediately post-intervention and at 6 months*). The primary outcome was functional health literacy skills (i.e., interpreting a thermometer, medicine, and food label) (**Table 2**). Secondary outcome measures were confidence, health literacy as measured by the Health Literacy Questionnaire (HLQ) (Osborne, Batterham, Elsworth, Hawkins, & Buchbinder, 2013), and patient activation (Hibberd, Mahoney, Stockard, & Tusler, 2005). At 6 months, additional measures (health knowledge, healthy lifestyle behaviors) were included. Shared decision-making was assessed as a measure of communicative and critical health literacy and reported in the companion article in this issue (Muscat et al., 2019)

### Statistical Analysis and Sample Size

Analyses were conducted using Stata version 14. Statistical significance was set at *p* < .05. Sample size calculations are detailed in full in the study protocol (McCaffery et al., 2016). Assuming unequal cluster sizes and allowing for up to 15% attrition, a total of 300 participants (150 per intervention group) would provide 90% power (with a two-sided alpha of .05) to detect differences in primary outcome measures between intervention groups as small as 0.5 standard deviations (*SD*).

Because of partial randomization, we tested for baseline differences between health literacy intervention and standard LLN arms using independent *t* tests or chi-squared tests for continuous and categorical measures, respectively. All outcome analyses were by intention-to-treat comparing the two arms using generalized estimating equations with an exchangeable correlation matrix and clustering by class group, adjusted for baseline health literacy (NVS) and baseline measure values (where available). For binary-dependent variables, logistic regression models were constructed. For continuous-dependent variables, linear regression models were used. Student satisfaction was analyzed using chi-squared statistics. Count data from the 6-month follow-up were analyzed using negative binomial regression due to overdispersion. Given the issues surrounding randomization, an *a priori* decision was made by the study investigators to conduct primary analyses collapsed across both randomized and nonrandomized classes (controlling for baseline values where available) to maximize sample size. The statisticians were blinded to the allocated group definitions until completion of primary analyses (see Appendix 2, available online at http://hdl.handle.net/2123/20364).

## Results

A total of 308 people across 10 TAFE NSW institutes (28 classes) participated in the trial; 167 in the health literacy arm, and 141 in the standard LLN arm (**Table 3**). Of these, 141 (46%) were randomized and the remaining 167 (54%) were not randomized. Responses to both baseline and immediate follow-up measures were available for at least one primary outcome measure from 116 (69%) health literacy participants and 97 (69%) standard LLN participants. Sample size varied slightly across measures due to missing data. As the baseline questionnaire was administered at three separate time points, in three separate class sessions (as described earlier), there are missing data due to student absence or late attendance. Missing data were not imputed. The difficulties of randomizing students and classes resulted in some baseline differences between intervention groups on demographic (language spoken at home, *p* = .003) and health literacy variables (NVS, *p* = .001; Single Item Literacy Screener, *p* = 0.04). To avoid multicollinearity issues, only differences in NVS were controlled for in regression models.

### Primary Outcomes: Functional Health Skills

A greater number of participants were able to interpret a thermometer after undergoing training in both groups (baseline vs. immediate follow-up, *p* = .001); however, there was little evidence that the health literacy and standard arms differed (*p* = .19) (**Table 4**). The proportion of participants able to accurately interpret medicine labels increased slightly in the health literacy arm; however, this was not significantly different from the standard arm (*p* = .19). Participants in the health literacy arm were significantly more accurate when interpreting a food label compared to those in the standard arm (*p* = .022). Change from baseline was not available for this measure.

### Secondary Outcomes

There was strong statistical evidence in both arms that participants' confidence in their own health skills increased after undergoing literacy training compared to baseline (*p* = .008); this change was significantly larger for those in the health literacy arm (*p* = .014) (**Table 4**).

*Communicative and critical health literacy.* Analysis of the HLQ subscales provided strong evidence of improvement in having sufficient information to manage health after taking part in literacy training in both groups (*p* = .004); however, the magnitude of change did not significantly differ between the health literacy and standard LLN arms (*p* = .14). After literacy training (health and standard LLN), participants reported significantly greater ability to actively manage their health (*p* = .017); there was evidence of this improvement being greater for those in the health literacy arm (*p* = .01) compared to the standard LLN arm. Small improvements at follow-up compared to baseline were observed for the other HLQ subscales assessed in the HL arm; however, these differences failed to achieve statistical significance. No significant difference was observed between trial arms on the Patient Activation Measure (*p* = .25).

### Student Satisfaction

**Figure 2.**
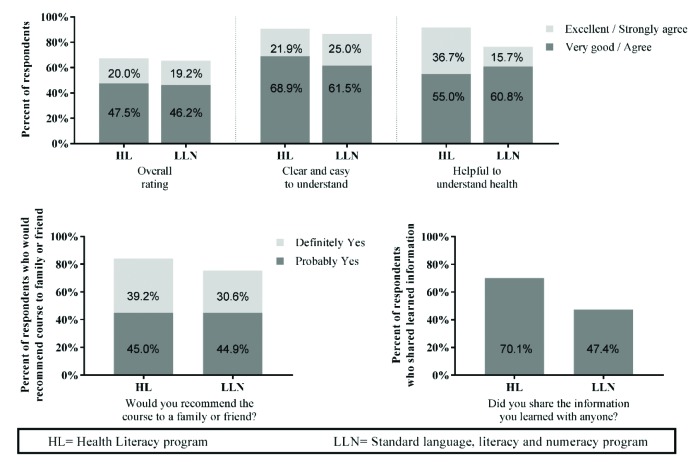
Student satisfcation scores. *HL = health literacy program; ^+^LLN = standard language, literacy, and numeracy program.

Overall satisfaction scores in both classes were high (**Figure 2**). Most students in the health literacy arm (67.5%) rated the program overall as *very good* or *excellent*; with similarly high levels reported in the standard group (LLN = 65.4%; *p* = .67). More than 90% of health literacy students agreed that the course was clear and easy to understand, with high levels also reported in the standard arm with no significant difference between groups (LLN = 86.5%; *p* = .59). In contrast, significantly more students in the health literacy arm *agreed/strongly agreed* that the course was helpful to understand their health than standard LLN students (91.7% vs. 77.5%; *p* = .007). There was no statistically significant difference between groups as to whether students would recommend the course to family or friends (84.2% vs. 75.5%; *p* = 0.54). However, a much greater proportion of students in the HL arm reported they shared the information with a family member or friend (70.1% vs. 47.4%; *p* = 0.011) compared to those in the standard arm.

### Six-Month Follow-Up

A total of 157 (51%) respondents completed and returned the follow-up questionnaire (**Table 5**). At 6 months, confidence in participants' own health skills remained elevated compared to baseline (mean improvement from baseline: 0.39, *p* < .001); however, there was little statistical evidence to suggest a difference between those in the health literacy and standard arms (*p* = .11). The health literacy arm had slightly higher health knowledge scores (68% correct vs. 60% correct), which was borderline statistically significant (*p* = .052). On the HLQ items, participants in both arms also reported a significantly greater ability to actively manage their health (*p* = .04), and to understand health information sufficiently to know what to do (*p* = .004) compared to baseline; however, the magnitude of change did not differ significantly across trial arms. Commensurate with analysis of immediate follow-up data, small and consistent but non-significant improvements were observed from baseline to 6-month follow-up across all other HLQ subscales. There were no significant differences in healthy lifestyle measures for diet and exercise.

## Discussion

This study demonstrated important health-related benefits of engaging adults in literacy programs, and the potential added benefits from incorporating health content. The study was able to recruit and retain socially disadvantaged, hard-to-reach adults from migrant and low educational backgrounds in Australia (NSW), and it is one of the few studies to report the impact of a health literacy-based adult education intervention at 6 months. The study showed both interventions were of benefit to students with statistically significant improvements from baseline to follow-up (immediate follow-up and 6 months) on several outcomes.

Improved performance among the health literacy intervention arm was observed across many functional, interactive, and critical health literacy outcomes. We found a consistent pattern of increased improvement compared to the standard literacy program, although only some outcomes reached statistical significance. This included reading a food label, confidence in health skills, and perceived ability to actively manage health (HLQ subscale) as well as health knowledge at 6 months. The health literacy course was rated more helpful in assisting students understand their health, and, notably, participants reported being more likely to share the information they learned with a family member or friend. Given that this is an identified hard-to-reach population, health literacy programs such as this may have potential for disseminating important public health messages into communities.

Data at 6 months indicated that learning was retained in both arms among students who responded (about 50%). Confidence in health skills, feeling able to actively manage your health, and understand health information sufficiently to know what to do remained significantly higher than baseline. There were no differences between arms on measures of health behavior; however, as a purely knowledge-based intervention this would have been unlikely. This is one of the first studies to report the impact of an adult education-based health literacy program at 6 months and suggests health literacy learnings are retained, which is encouraging.

By contrast, a recent study from Taiwan reported no differences in health literacy at 6 months but reduced emergency department visits and hospitalizations (Tsai, Lee, & Yu, 2018). However, our findings are consistent with studies that integrate health literacy into ESL programs that show improvements in knowledge and functional health literacy (Chen et al., 2015; Soto Mas, Ji, Fuentes, & Tinajero, 2015; Soto Mas, Schmitt, Jacobson, & Myers, 2018). Earlier studies (Elder et al., 1998; Elder et al., 2000 reported changes in cardiovascular risk factors at 3 months after a health literacy intervention, although these changes were not sustained at 6 months. We did not find differences in health behavior between our standard and health literacy arms and did not take baseline measures, so we cannot say whether the intervention led to an improvement across both groups. Future studies using fully randomized designs need to examine further how health literacy interventions affect health-seeking behavior, health service use, and interactions with health care professionals. Our results also concur with the evaluation of the UK Skilled for Health Program, which found improved self-reported outcomes and strong engagement and interest in the health literacy program among students (Tavistock Institute and Shared Intelligence, 2009).

Our study is unusual in that it compares a standard LLN program against a context-specific program in health and examines the affect on health literacy outcomes. This design sheds light on the debate about transferable skills (analysis, planning, problem-solving) versus contextualized learning in which acquired skills are specific to the activity and domains of knowledge in which they were learned (Cromley, 2000). Our study suggests that students in the LLN program acquired literacy and numeracy skills that they could transfer into a health setting and showed improvements on health literacy measures from baseline to follow-up. This is encouraging for LLN programs everywhere and indicates a potential benefit for health of general literacy programs. However, our study showed that outcomes were better in the health literacy arm at both immediate and 6-month follow-up, so if the objective is to improve health literacy then a contextualized program is optimum.

## Strengths and Limitations

The strengths of this study include successful intervention delivery and data collection with a diverse, socially disadvantaged adult population with measures at baseline, immediately post-intervention, and at 6 months across a comprehensive set of health literacy outcomes. The study represents an important cross-sectoral collaboration between health and education partners, including the state health department, national and state-based health organizations, and the government-funded national provider of adult education. The program was delivered by trained adult educators in TAFE colleges and continues to be taught in some colleges in NSW, meaning it is sustainable and potentially scalable.

The major limitation of the study was the challenge with randomization among a subset of the participating colleges. We sought to address this by providing analyses on both randomized-only and the full sample data and adjusting for baselines differences between intervention arms. We also had missing data due to some students experiencing difficulties completing all questionnaire items within the allocated class time and student absences from class when assessments took place. Our final 6-month assessment included only 50% of participants. However, as our sample is relatively transient and TAFE colleges underwent significant structural changes over the duration of the study (which subsequently required students to pay fees for tuition), it is not surprising that our follow-up rate was not higher. We did not conduct any analysis of the impact of the intervention on different demographic subgroups. This is because the study was not powered to conduct subanalyses by demographic variables (McCaffery et al., 2016), and any interpretation of underpowered post-hoc sub analyses would be difficult or misleading. Future studies should be designed to target demographic subgroups and appropriately powered to investigate intervention effects.

## Conclusion

The study shows that improving LLN skills has potential public health benefits that are retained at 6 months without further reinforcement. This project demonstrated valuable additional benefits from integrating specific health content into adult basic learning and demonstrated this is feasible and potentially scalable. The program was rated positively by a high-risk, socially disadvantaged population of students, producing added health literacy benefits to the standard LLN curriculum that were retained over time. Teachers also rated the course highly and reported it was easy to deliver and engaged students in learning (Muscat et al., 2019). The program highlights the value of adult education in the community and the added value that can be achieved by cross-sectoral collaboration between education and health in improving health literacy and public health in the community.

## Figures and Tables

**Table 1:** Topics Covered in the Intervention

**Being Healthy (Teacher Manual 1)**	**Staying Healthy (Teacher Manual 2)**
Taking temperature^a^	Getting involved
Checking medicine labels^a^	Food groups
Prescriptions	Food labels^a^
Dosage and timing	Nutritional information^a^
Health workers	Food temperature safety
Telling your doctor what is wrong^a^	Food date safety
Talking to your doctor^a^	What is a serving^a^
Answering your doctor's questions^a^	Budgeting
Immunization and health screening	Understanding a diet
Asking questions^a^	Drinking enough fluids
Shared decision-making^a^	Heart rate and pulse
Completing medical forms	Being active
Emergency services	Watch first aid demonstrations
Advice from pharmacist	Follow written instructions
Saving lives	Talking on the telephone^a^
Follow emergency instructions	Revision/goal setting

a Core topics that all students were required to study to complete the course. The remaining topics were optional and decided by students and teachers for inclusion based on interest and relevance for the group.

**Table 2:** Study Measures

**Outcome Measure**	**Description**	**Time of Measurement**
Functional health skills	Reading a thermometer,^a^ interpreting a medicine label,^a^ and a food label (McCaffery et al., 2016; Muscat et al., 2016) Skills were assessed separately, and as a combined outcome, weighted by the sum of the components	T0, T1
Confidence in health skills	10 confidence items, measured on a 5-point Likert-scale ranging from *extremely* to *not at all* confident (Chew, Bradley, & Boyko, 2004)	T0, T1, T2
Communicative and critical health literacy	Health Literacy Questionnaire subscales: we used five relevant subscales: (1) having sufficient information to manage my health, (2) actively managing my health, (3) ability to engage with health care providers, (4) navigating the health care system, (5) understanding health information well enough to know what to do (Osborne et al., 2013)	T0, T1, T2
Critical health literacy	Patient activation measure (Hibberd et al.; 2005)	T1
Student satisfaction	Students rated course experience using 5-point Likert scale items for these three criteria: (1) overall rating; (2) if the course was (a) easy to understand or (b) helped them to understand their health; (3) if they would recommend the course to family and friends	T1
Health knowledge	12-item curriculum-based measure to assess retention of core components of program (McCaffery et al., 2016)	T2
Health behavior	Self-report items of daily fruit and vegetable intake (number of servings), daily walking, and moderate and hard physical activity (Sax Institute, 2015)	T2

Note. T0 = baseline; T1 = immediately post-intervention; T2 = 6 months.

a Indicates item was assessed at baseline.

**Table 3:** Demographic Characteristics of the Sample with Both Baseline and Immediate Follow-Up Data on at Least One Primary Outcome Measure

**Variable [Score Range]**	**Health Literacy Arm**		**Standard LLN Arm**			**All Participants**	
			
	***n***	**M (*SD*) or %**	***n***	**M (*SD*) or %**	***p* Value**	***n***	**M (*SD*) or %**

Age (years)	116	47.3 (13.5)	97	47.6 (15.8)	.90	213	47.4 (14.5)

Gender (female)	79	68	70	73	.45	149	70

Country of birth							
Australia	31	27	42	43	.06	73	34
China	22	19	17	18		39	18
Vietnam	4	3	4	4		8	4
Other^a^	59	51	34	35		93	44

Highest level of schooling completed (or equivalent)							
Year 11 or 12	42	39	35	38	.88	77	39
Year 10	19	18	20	22		39	20
Year 9	17	16	17	19		34	17
Year 8	26	24	18	20		44	22
No formal schooling	3	3	2	2		5	3

Residential region (metropolitan/regional)							
Metropolitan	80	69	55	57	.06	135	63

Primary language spoken at home							
English	31	30	46	51	.003	77	39

Longstanding illness/disability							
Yes	78	68	56	58	.15	134	64

Baseline HL							
Newest Vital Sign							
Inadequate^b^	94	81	58	60	.001	152	71
Self-rated reading ability							
Limited HL^c^							
Single Item	62	63	49	55	.25	111	59
Literacy Screener							
Limited HL^d^	81	81	62	68	.04	143	67

Note. HL = health literacy; LLN = language, literacy, and numeracy.

a Category includes countries of birth for which the total frequency was less than eight participants or was not specified.

b Inadequate or limited health literacy designated by scoring ≤3 on the Newest Vital Sign.

c Inadequate or limited health literacy designated by responding with *okay* or worse on self-rated reading ability.

d Inadequate or limited health literacy designated by responding with *sometimes* on the single-item literacy screener.

**Table 4:** Descriptive Statistics for Primary and Secondary Outcomes for Participants Included in Adjusted Analyses of Immediate Follow-Up Data Only^a^

**Variable [Score Range]**	**Health Literacy Arm**		**Standard LLN Arm**		**Absolute Difference**	***p* Value**	
		
	***n***	**M (*SD*) or %**	***n***	**M (*SD*) or %**		**Comparison**	**Change from Baseline**

Functional health skills							

Reading a thermometer (pass)							
Baseline	109	42	84	47.6	−5.4%		
Immediate follow-up		60.6		54.8	5.8%	.19	.001
Change from baseline		18.4		7.2	11.2%		

Medicine label (pass)							
Baseline	111	27	91	40.7	−13.7%		
Immediate follow-up		36		39.6	−3.6%	.19	.28
Change from baseline		7		−1.1%	8.1%		

Food label [0–10]							
Immediate follow-up	115	6.03 (2.34)	96	5.49 (2.30)	0.54	.022	–

Secondary outcomes							

Confidence [1–5]							
Baseline	111	2.47 (0.69)	91	2.66 (0.81)	0.19		
Immediate follow-up		2.81 (0.61)		2.72 (0.76)	0.09	.014	.008
Change from baseline		0.34		0.06			

PAM-13 [0–100]							
Immediate follow-up	109	56.6 (15.1)	64	54.9 (18.1)	1.7	.25	–

HLQ/having sufficient information to manage health [1–4]							
Baseline	105	2.80 (0.49)	60	2.9 (0.46)	−0.10		
Immediate follow-up		3.03 (0.46)		2.99 (0.50	0.04	.14	.004
Change from baseline		0.23		0.09	0.14		

Actively managing health [1–4]							
Baseline	105	2.84 (0.49)	59	2.89 (0.56)	−0.05		
Immediate follow-up		2.97 (0.43)		2.95 (0.46)	0.02	.01	.017
Change from baseline		0.13		0.06	0.07		

Active engagement with health care providers [1–5]							
Baseline	103	3.50 (0.77)	57	3.74 (0.68)	−0.24		
Immediate follow-up		3.67 (0.69)		3.76 (0.70)	−0.09	.38	.10
Change from baseline		0.17		0.02	0.15		

Navigating the health care system [1–5]							
Baseline	103	3.54 (0.66)	57	3.65 (0.66)	−0.11		
Immediate follow-up		3.69 (0.70)		3.65 (0.69)	−0.04	.13	.19
Change from baseline		0.15		0	0.15		

Understanding health information enough to know what to do [1–5]							
Baseline	103	3.34 (0.72)	59	3.68 (0.72)	−0.34		
Immediate follow-up		3.60 (0.72)		3.70 (0.69)	−0.10	.12	.15
Change from baseline		0.26		0.02	0.24		

Note. HLQ = health literacy questionnaire; LLN = language, literacy, and numeracy program; PAM = patient activation measure.

a Analyses were adjusted for baseline health literacy and baseline values of each measure (where available) clustered by class group.

**Table 5:** Descriptive Statistics of 6-Month Outcome Measures for Participants Included in the Analyses, Adjusted for Baseline Health Literacy and Baseline Values of Each Measure, Where Available, Clustered By Class Group

**Variable [Score Range]**	**Health Literacy Arm**		**Standard LLN Arm**		**Absolute Difference**	***p* Value**	
		
	***n***	**M (*SD*) or %**	***n***	**M (*SD*) or %**		**Comparison**	**Change from Baseline**

Confidence [1–5]							
Baseline	78	2.44 (0.73)	63	2.57 (0.88)	−0.13		
6-month follow-up		2.91 (0.76)		2.86 (0.75)	0.05	.11	<.001
Change from baseline		0.47		0.29	0.18		

Health literacy questionnaire							

Having sufficient information to manage health [1–4]							
Baseline	76	2.83 (0.52)	56	2.84 (0.49)	−0.01		
6-month follow-up		3.03 (0.55)		2.93 (0.38)	0.10	.23	.13
Change from baseline		0.20		0.09	0.11		

Actively managing health [1–4]							
Baseline	76	2.86 (0.50)	55	2.94 (0.56)	−0.08		
6-month follow-up		3.01 (0.55)		3.01 (0.45)	0.00	.79	.04
Change from baseline		0.15		0.07	0.08		

Active engagement with health care providers [1–5]							
Baseline	75	3.55 (0.79)	54	3.59 (0.81)	−0.04		
6-month follow-up		3.66 (0.73)		3.70 (0.65)	−0.04	.99	.07
Change from baseline		0.11		0.11	0.00		

Navigating the health care system [1–5]							
Baseline	75	3.55 (0.69)	55	3.51 (0.77)	0.04		
6-month follow-up		3.65 (0.75)		3.59 (0.74)	0.06	.76	.15
Change from baseline		0.10		0.08	0.02		

Understanding health information enough to know what to do [1–5]							
Baseline	75	3.28 (0.77)	55	3.50 (0.72)	−0.22		
6-month follow-up		3.59 (0.79)		3.60 (0.69)	0.01	.36	.004
Change from baseline		0.31		0.10	0.21		

Healthy lifestyle							

Meets guidelines for recommended fruit intake (2 servings per day)							
6-month follow-up	78	69.2	65	73.9	−4.7%	.69	–

Meets guidelines for recommended vegetable intake (5 servings per day)							
6-month follow-up	78	19.2	63	20.6	−1.4%	.82	–

Walking for at least 10 minutes (number of sessions per week)							-
Median (IQR)	72	4 (2–7)	59	5 (2–7)	−1	.94	

Vigorous physical activity for at least 20 minutes (number of sessions per week)							
Median (IQR)	75	0 (0–3)	61	1 (0–3)	−1	.81	-

Moderate physical activity for at least 30 minutes (number of sessions per week)							
Median (IQR)	74	21 (1–3)	62	1 (0–3)	1	.19	-

Health knowledge							

Health and servings quiz [0–12]	61	8.16 (2.16)	54	7.24 (2.49)	0.92	.052	-

Note. IQR = interquartile range.
